# Adherencia al tratamiento y personalidad resiliente en mayores de 65 años polimedicados

**DOI:** 10.1016/j.aprim.2025.103343

**Published:** 2025-07-21

**Authors:** María Esther Cortes-Fernandez, Eva Cortes-Fernandez, Emilio García-Jimenez, María José Zarzuelo

**Affiliations:** a Universidad de Granada, Granada, España; b Distrito Centro de Almería, Almería, España; c Vocalía de farmacia de la Sociedad Andaluza de Hipertensión y Riesgo Cardiovascular, Granada, España

En España el 19% de la población supera los 65 años y una proporción considerable presenta polimedicación^1^. Esta situación puede derivar en una baja adherencia terapéutica, lo que incrementa el riesgo de reacciones adversas, complicaciones clínicas y un mayor gasto sanitario^2^, ^3^. En este contexto la psicología de la salud ha identificado variables personales que podrían actuar como factores protectores. Entre ellas destaca la personalidad resiliente, que integra 3 dimensiones: compromiso, control y desafío^4^. Las personas con altos niveles de personalidad resiliente tienden a afrontar el estrés de forma activa y a adoptar conductas más saludables^4^, ^5^. Comprender esta relación podría facilitar el diseño de intervenciones psicológicas centradas en mejorar los rasgos de personalidad que favorecen la adherencia, y con ello, optimizar el tratamiento y bienestar de este grupo vulnerable.

El objetivo de este estudio fue analizar la relación entre la personalidad resistente y la adherencia terapéutica en personas mayores de 65 años con polimedicación.

Se empleó un diseño de investigación no experimental, de tipo *ex post facto* con enfoque correlacional.

Los participantes eran residentes de una zona rural de Almería. Los criterios de inclusión fueron: tener 65 años o más y disponer de autonomía funcional para responder a los cuestionarios. Los criterios de exclusión fueron: presencia de deterioro cognitivo que impidiera la comprensión de las preguntas.

Los instrumentos usados fueron el Cuestionario de adherencia al tratamiento (Morisky-Green) y el Cuestionario de personalidad resistente, adaptado por Moreno-Jiménez et al.^6^. La recogida de datos se realizó en la zona de atención personalizada de la farmacia comunitaria de Almería, del 1 al 30 de septiembre de 2024.

Para el análisis de datos se utilizó el programa estadístico SPSS (versión 29). Se realizaron análisis descriptivos (frecuencias, porcentajes, medias, desviaciones estándar) y pruebas inferenciales, incluyendo Chi-cuadrado de Pearson para relaciones entre variables cualitativas y pruebas de comparación de proporciones con nivel de significación α = 0,05.

De los 110 participantes el 60,9% (n = 67) estaba clasificado como polimedicado, y tenía 73,0 ± 4,6 años. De ellos, el 65,6% eran mujeres y el 83,6% solo habían completado la educación primaria. El 75,5% (n = 83) era adherente al tratamiento según los criterios de Morisky-Green. En cuanto a la personalidad resiliente, el 57,3% (n = 63) puntuaron en un nivel medio, el 33,6% (n = 37) en un nivel alto y el 9,1% (n = 10) en nivel bajo (tabla 1).

**Tabla 1:** Datos sociodemográficos y relación entre el nivel de personalidad resiliente y la adherencia terapéutica con el género y el nivel de estudios

	N	%
Edad (media ± DE; años)	73,0	4,6
Género (femenino)	72	65,6
Educación (hasta estudios primarios)	92	83,6
Polimedicado (sí)	67	60,9
Adherencia terapéutica (sí)	83	75,5
Nivel de personalidad resiliente (alta)	37	33,6

	Nivel de personalidad resiliente		Adherencia terapéutica		
	Bajo n (%)	Medio n (%)	Alto n (%)	No adherente n (%)	Adherente n (%)
*Adherencia terapéutica*					
No adherente	6 (100)	15 (44,1)	1 (3,7)	-	-
Adherente	0 (0)	19 (55,9)	26 (96,3)	-	-
		(Chi cuadrado = 24,63; p < 0,001)			
*Género*					
Masculino	1 (16,7)	10 (29,4)	7 (25,9)	9 (40,9)	9 (20,0)
Femenino	5 (83,3)	24 (70,6)	20 (74,1)	13 (59,1)	36 (80,0)
		(Chi cuadrado = 0,44; p = 0,802)		(Chi cuadrado = 3,29; p = 0,070)	
*Nivel de estudios*					
Hasta estudios primarios	9 (90,0)	54 (85,7)	29 (78,4)	27 (100,0)	65 (78,3)
Estudios secundarios y universitarios	1 (10,0)	9 (14,3)	8 (21,6)	0 (0,0)	18 (21,7)
		(Chi cuadrado = 5,12, p = 0,529)		(Chi cuadrdo = 11,83; p < 0,008)	

DE: desviación estándar.

Se reveló una asociación estadísticamente significativa entre la personalidad resiliente y la adherencia (Chi cuadrado = 24,63; p < 0,001). Entre los participantes con altos niveles de personalidad resiliente el 96,3% eran adherentes al tratamiento, con una fuerte relación de fuerza de asociación (V de Cramér = 0,627) (tabla 1).

Aunque las mujeres mostraron una mayor tasa de polifarmacia (68,1%), no se encontraron diferencias significativas entre el género en la adherencia (Chi cuadrado = 3,29; p = 0,070) ni en los niveles de personalidad resiliente (Chi cuadrado = 0,44; p = 0,802) (tabla 1).

Se reveló una asociación significativa (Chi cuadrado = 11,83; p = 0,008), con una asociación lineal significativa (9,02; p = 0,003) a través de las categorías educativas y la adherencia terapéutica. La adherencia fue mayor entre los participantes con estudios primarios (78,3%), con una asociación moderada (V de Cramér = 0,429) (tabla 1).

## Conclusiones

Adultos mayores de 65 años polimedicados con altos niveles de compromiso, control y desafío tienden a mostrar conductas más saludables y un afrontamiento más eficaz del estrés y la enfermedad.

Estos datos ponen de relieve la necesidad de intervenciones personalizadas que no solo consideren el número de medicamentos o las instrucciones clínicas, sino también las características psicológicas y sociales del paciente, entre ellas la personalidad resiliente.

## Financiación

Este estudio no ha recibido financiación.

## Consideraciones éticas

El procedimiento fue aprobado por el Comité de Ética del Hospital Universitario Torrecárdenas, con código 10012025. Cada paciente que participó en el estudio firmó un consentimiento informado.

## Conflicto de intereses

Los autores declaran que no existe conflicto de intereses.
